# Production and Characterization of a Novel, Electrospun, Tri-Layer Polycaprolactone Membrane for the Segregated Co-Culture of Bone and Soft Tissue

**DOI:** 10.3390/polym8060221

**Published:** 2016-06-07

**Authors:** Sasima Puwanun, Frazer J. Bye, Moira M. Ireland, Sheila MacNeil, Gwendolen C. Reilly, Nicola H. Green

**Affiliations:** 1 Faculty of Dentistry, Naresuan University, Phitsanulok 65000, Thailand; sasimap@nu.ac.th; 2 Kroto Research Institute, University of Sheffield, Sheffield S3 7HQ, UK; frazer.bye@gmail.com (F.J.B.); maighread29@hotmail.com (M.M.I.); s.macneil@sheffield.ac.uk (S.M.); 3 INSIGNEO Institute for in silico Medicine, University of Sheffield, Sheffield S10 2TN, UK; g.reilly@sheffield.ac.uk

**Keywords:** mesenchymal stem cells, maxillofacial surgery, bone tissue engineering, soft tissue engineering, scaffold, electrospinning

## Abstract

Composite tissue-engineered constructs combining bone and soft tissue have applications in regenerative medicine, particularly dentistry. This study generated a tri-layer, electrospun, poly-ε-caprolactone membrane, with two microfiber layers separated by a layer of nanofibers, for the spatially segregated culture of mesenchymal progenitor cells (MPCs) and fibroblasts. The two cell types were seeded on either side, and cell proliferation and spatial organization were investigated over several weeks. Calcium deposition by MPCs was detected using xylenol orange (XO) and the separation between fibroblasts and the calcified matrix was visualized by confocal laser scanning microscopy. SEM confirmed that the scaffold consisted of two layers of micron-diameter fibers with a thin layer of nano-diameter fibers in-between. Complete separation of cell types was maintained and calcified matrix was observed on only one side of the membrane. This novel tri-layer membrane is capable of supporting the formation of a bilayer of calcified and non-calcified connective tissue.

## 1. Introduction

An important clinical goal in tissue engineering is to move beyond the reconstruction of single tissue types to generate composite tissues, composed of two or more tissue types, which more closely reflect the basic unit for healing *in vivo*. This is of particular significance in several clinical settings, including cleft lip/cleft palate repair, trauma surgery and tumor removal. However, to date, only a small number of studies have reported success in generating a scaffold capable of generating distinct tissue layers for both soft tissue and bone repair. A suitable scaffold should allow for normal development of both soft tissue and the slower growing bone tissue, while preventing soft tissue intrusion into the bone formation site. The scaffold should also permit a good flow of nutrients throughout the scaffold to facilitate cell proliferation. Electrospinning was selected for scaffold production due to the level of control available in fiber size, fiber orientation and porosity [1].

Mesenchymal stem cells (MSCs) have been used for bone regeneration due to their ability to differentiate into osteoblasts, osteocytes and adipocytes and have previously shown satisfactory clinical outcomes [2,3]. Human embryonic cell derived mesenchymal progenitor cells (hES-MPs) [4] and primary human MSCs derived from bone marrow mononuclear cells were employed in this proof-of-concept study as model cells for bone tissue. Primary human dermal fibroblasts were chosen to represent the soft tissue layers.

The first aim of this preliminary study was to generate an electrospun tri-layer scaffold using only one polymer, poly-ε-caprolactone (PCL). The second was to seed the tri-layer scaffold with cells capable of generating bone and soft tissue, demonstrate localized mineral deposition and evaluate the extent to which the scaffold could maintain spatial separation of the two layers.

## 2. Materials and Methods

All products are from Sigma Aldrich, Dorset, UK unless otherwise stated.

### 2.1. PCL Scaffold Fabrication

10 wt % solution PCL (molecular number 80,000) in dichloromethane (DCM) was used for microfiber production, and 5 wt % solution PCL in methanol:DCM mixture (weight ratio 10.5:89.5) for nanofiber production, stirred for 24 h at room temperature. Scaffolds were fabricated with an electrospinning rig, at room temperature, as previously described [1,5], with one rotating drum collector (126 mm diameter) and two syringe pumps, each with four needles, located on either side of the collector. Fibers were fabricated at 17 kV, 40 µL/min flow rate, 300 revolutions/min drum rotation speed. Microfibers and nanofibers were generated from the two pumps at 17 and 10 cm from the collector respectively. 8 mL 10 wt % PCL solution was dispensed from the outer pump to generate the initial microfiber layer, followed by simultaneous release of 4 mL solution from this pump and 4 mL 5 wt % PCL solution from the inner pump to generate the intervening nanofiber layer. The second microfiber layer was generated in the same manner as the initial layer, dispensing 8 mL solution from the outer pump. Scaffolds were allowed to dry at room temperature and cut using a circular cork borer (15 mm diameter), prior to sterilization with 0.1 *v*/*v* % peracetic acid in PBS.

### 2.2. Scanning Electron Microscopy (SEM) Characterization of Scaffolds

Scaffolds without cells were prepared for SEM (Phillips XL-20 SEM, Eindhoven, The Netherlands) by coating with an ultrathin gold layer. The pore size and fiber diameter (*n* = 100) were analyzed from four recorded SEM micrographs in each layer (width 200 µm each layer) using ImageJ, (National Institutes of Health, Bethesda, MD, USA) [6]. Each picture was overlaid by a square grid, size 900 µm^2^, and the diameter determined for pores and fibers underneath the junctions of the squares. To determine pore size area, a pixel bandpass filter was applied (minimum 3 pixels, maximum 20 pixels), the images thresholded and the void space determined.

Cell-seeded scaffolds were removed from culture media, PBS washed ×3 and fixed (10 *v*/*v* % formalin in PBS, 20 min, room temperature). Scaffolds were dehydrated by sequentially submerging (15 min) in 35%, 60%, 80% and 90% *v*/*v* ethanol in distilled water, followed by 100% ethanol. Samples were submerged in 1:1 ethanol:hexamethyldisilazane (HDMS) solution for 1 h, rinsed ×2 in 100% HDMS for 5 min and dried in a dessicator for at least 1 h prior to gold-coating.

### 2.3. Cell Culture

hES-MPs (Cellartis, Gothenburg, Sweden) were cultured in α-MEM supplemented with 10 *v*/*v* % fetal calf serum (FCS), 2 mM l-glutamine, 100 mg/mL penicillin, streptomycin (P/S), and 4 ng/mL fibroblast growth factor-basic recombinant human (bFGF: Invitrogen, Paisley, UK) in 1% gelatin pre-coated flasks. hBMSCs were isolated from mononuclear cells (Lonza, Basel, Switzerland); briefly; mononuclear cells were transferred to basal culture media containing 0.1 mg/mL DNaseI (Stemcell Technologies, Cambridge, UK) and plated (1.2 × 10^5^ cells/cm^2^). After 7 days non-adherent cells were removed. The adherent cells (passage 0) were cultured in expansion media (EM—α-MEM, 10 *v*/*v* % FCS, 2 mM l-glutamine, 100 mg/mL P/S) until 70%–80% confluent. hBMSC cells were seeded on scaffolds at passage 2 or 3.

Following cell inclusion on scaffolds, samples were cultured in supplementary media (SM), EM supplemented with 50 µg/mL ascorbic acid-2-phosphate (AA) and 5 mM β-glycerolphosphate (β-GP), or osteogenic induction media (OIM—SM with dexamethasone). The levels of dexamethasone were optimized in previous studies (100 nM Dex for hES-MP cells [7], 10 nM for hBMSC cells [8]).

Fibroblasts were isolated from human dermis as previously described [4] (HTA license 12179). The fibroblasts were cultured in DMEM, 10 *v*/*v* % FCS, 2 mM l-glutamine, 100 IU/mL P/S, 0.625 µg/mL amphotericin B and used between passages 3 and 5.

### 2.4. Assessment of Cell Viability of hES-MPs on Electrospun PCL Scaffold

Scaffolds were placed in a CellCrown^TM^ assembly. 1 mL basal media was added, 10^5^ cells in 100 µL media seeded on top of the scaffolds and left for 24 h in an incubator. The scaffolds were removed to fresh wells containing SM or OIM culture media. Media was replaced every 2 to 3 days and culture continued for 7, 14, 21 or 28 days. At the end the media was replaced with 0.1 mM resazurin salt solution in basal media and incubated (4 h, 37 °C). Two hundred microliters of media was removed and the concentration of resorufin determined with a spectrofluorometer (λ_ex_ 540 nm, λ_em_ 630nm).

### 2.5. hES-MPs Cell Line Seeding and Proliferation on Tri-Layer Electrospun PCL Scaffold

Preliminary experiments were carried out using the hES-MPs cell line. Scaffolds were seeded with 1 × 10^5^ hES-MPs (100 µL media) in a CellCrown^TM^ assembly and grown in OIM, for 28 days. Scaffolds were 10 *v*/*v* % formalin-fixed, permeabilised with 1% Triton X-100 and stained with 4′-6-diamidino-2-phenylindole (DAPI). For actin staining, cells were incubated with 2 µg/mL Phalliodin TRITC for 15 min prior to washing. Cell nuclei (Ti-Sapphire two-photon laser λ_ex_ 800 nm, λ_em_ 435–485 nm) and actin (λ_ex_ 543 nm, λ_em_ 565–615 nm) were visualized. Differential interference contrast (DIC) imaging was used to locate the scaffold.

### 2.6. Co-Cultured between hBMSCs and Fibroblasts on Tri-Layer Electrospun PCL Scaffold

Scaffolds were co-cultured with hBMSCs and human dermal fibroblasts. 1 × 10^5^ hBMSCs (100 µL media) were seeded in a CellCrown^TM^ and cultured in OIM for 21 days. The CellCrown^TM^ was then inverted and 1 × 10^5^ fibroblasts (100 µL media), stained with CellTracker^TM^Green (Invitrogen, Paisley, UK), added to the opposite side of the scaffold. The entire construct was cultured, without dexamethasone to minimize the risk of mineralization of the fibroblast layer [7], for 7 days. At the end of the culture period, xylenol orange (C_31_H_28_N_2_O_13_SNa_4_) was used to indicate mineralization. The scaffold was incubated in media containing XO (20 µM) for 12 h. The scaffolds were detached from the CellCrown^TM^ inserts, cut into 0.5 mm strips, orientated with the cut surface uppermost to facilitate side-on viewing, and imaged with an LSM 510Meta upright microscope (Carl Zeiss, Cambridge, UK) (λ_ex_ 543 nm, λ_em_ 565–615 nm). The location of CellTracker^TM^ Green-stained fibroblasts was also determined (λ_ex_ 488 nm, λ_em_ 500–550 nm). Images (512 × 512) were obtained using a 20×, 0.5 NA, EC Plan-Neofluar water-dipping objective. For co-culture experiments without the barrier layer fibroblasts were labelled with CellTracker^TM^Green as above and hES-MPs with CellTracker^TM^Red (Invitrogen, Paisley, UK) (λ_ex_ 543 nm, λ_em_ 565–615 nm) prior to incorporation into the scaffold.

## 3. Results and Discussion

### 3.1. PCL Tri-Layer Scaffold Fabrication

**Figure 1:**
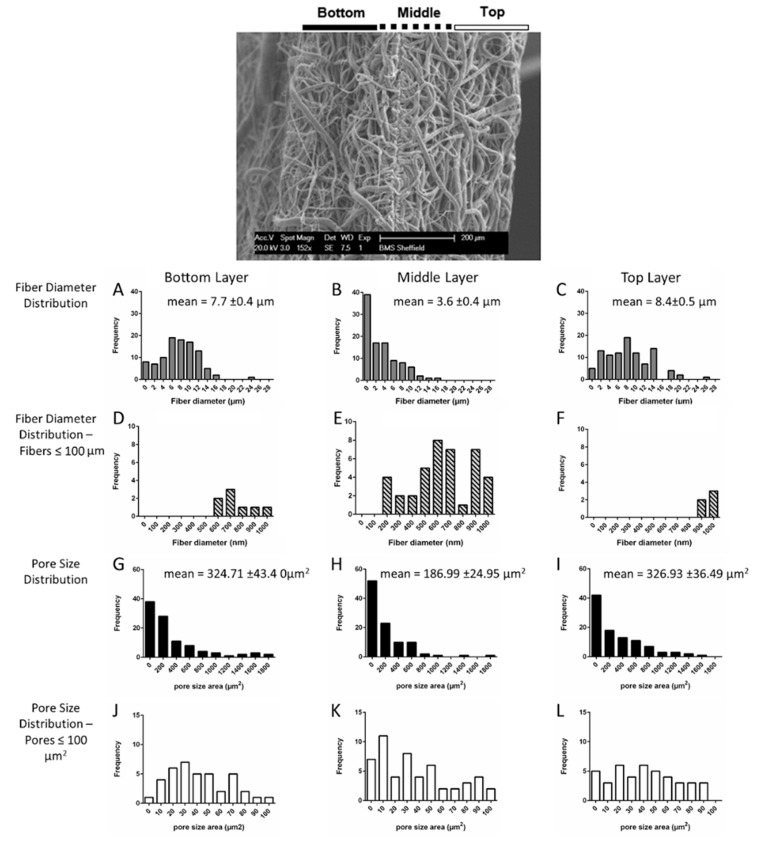
SEM image of the tri-layer electrospun PCL scaffold in side view (top), scale bar: 200 µm. Overall fiber diameter distribution (**A**–**C**) and the distribution specifically below 100 µm (**D**–**F**) in the bottom (**A**,**D**), middle (**B**,**E**), and top (**C**,**F**) layers; overall fiber pore size distribution (**G**–**I**) and the distribution specifically below 100 µm^2^ (**J**–**L**) in the bottom (**G**,**J**), middle (**H**,**K**) and top (**I**,**L**) layers (*n* = 100 from 4 SEM images per layer, mean ± SE).

Scaffold structure and fiber diameters were characterized prior to cell seeding. Differences were observed in the fiber size distribution throughout the layers (Figure 1). In the bottom layer >90% fibers were microfibers, *i.e.*, >1 µm (mean 7.7 ± 0.4 µm, Figure 1A,D). In the middle layer >40% were nanofibers, ≤1 µm (mean 3.6 ± 0.4 µm Figure 1B,E). In the top layer >95% were microfibers (mean 8.4 ± 0.5 µm, Figure 1C,F). Fiber diameter is directly related to polymer concentration, however low concentration solutions form fibers with beads [9]. The addition of alcohol can greatly reduce bead formation due to its higher permittivity [10]. Therefore, 5% PCL in methanol/DCM was used for nanofiber and 10% PCL in DCM for microfiber production. The pore size also varied between the nano- and microfiber layers (Figure 1G–L), with ~325 µm^2^ mean pore size for the two microfiber layers. Figure 1J–L shows the frequency distribution of the pores with an area ≤100 µm^2^. It showed that in the upper and lower regions of this layer 29% and 28% of the pores were of 50 µm^2^ or less, and in the middle layer 40% of these pores were of 50 µm^2^ or less suggesting that the middle layer could act as a cell barrier since this is below the minimum pore size required for cell infiltration.

### 3.2. Progenitor Cell Seeding and Proliferation on Tri-Layer Electrospun PCL Scaffolds

**Figure 2:**
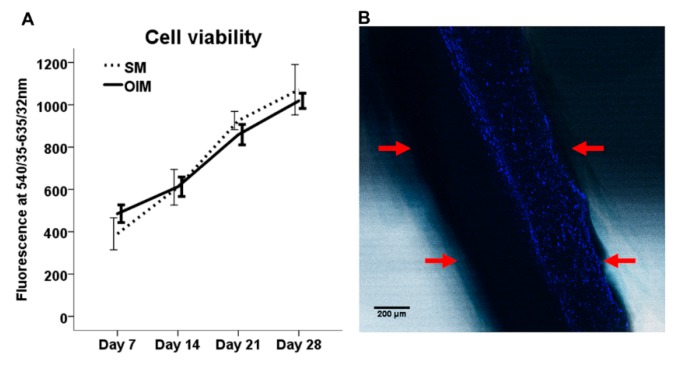
(**A**) Cell viability of hES-MPs on single layer electrospun PCL scaffolds with SM and OIM media, measured after 7, 14, 21, and 28 days, mean ± SE, *n* = 6; (**B**) Tri-layer scaffold, located by DIC imaging, seeded with hES-MPs, labeled with DAPI (blue), showing no cells crossed the center of the scaffold in 28 days. Red arrows indicate the edge of the scaffold (scale bar = 200 µm).

Preliminary studies to assess the response of osteogenic cells to the scaffold were performed using hES-MPs. These have been used as a model cell line for bone tissue engineering [11]. The viability of hES-MPs increased over time for 28 days in both SM and OIM media on single layer PCL scaffolds (Figure 2A), demonstrating dexamethasone has no effect on cell proliferation. On tri-layer scaffolds, all cells were located on the same side of the scaffold on which they were seeded, with no evidence of cells crossing over the central nanofiber region (Figure 2B), supporting our hypothesis that cells are unable to penetrate the nanofibrous layer due to its low porosity and demonstrating that the tri-layer scaffolds can support hES-MP attachment and proliferation.

### 3.3. Co-Culture between hBMSCs and Fibroblasts on Tri-Layer Electrospun PCL Scaffolds

hBMSCs were used in subsequent tri-layer experiments as they are more clinically relevant. To ascertain the behavior of both osteogenic and soft tissue forming cells on the same scaffold, they were co-cultured with dermal fibroblasts. Xylenol orange was used to reveal osteogenic cell mineralization, as it binds to newly laid down bone mineral [12]. This has been used for the visualization of mineralized tissue in 3D scaffolds [13]. Primary human dermal fibroblasts represent the soft tissue layer and oral fibroblasts can also be extracted from the buccal mucosa and used to generate soft tissue [14].

**Figure 3:**
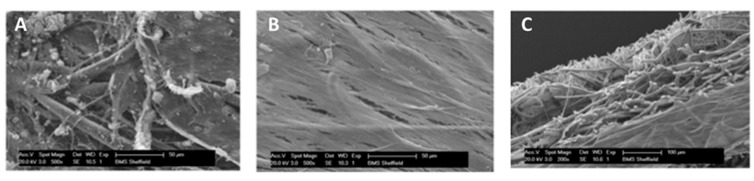
SEM face (**A**,**B**—scale bar = 50 µm) and side (**C**—scale bar = 100 µm) views of the tri-layer scaffold following co-culture with hBMSCs and fibroblasts. Matrix mineralization is observed on one side of the scaffold (**A**) and soft tissue on the other (**B**).

SEM imaging revealed calcium nodules on the hBMSCs side (Figure 3A). Fibroblasts were clearly present in a dense layer on the opposing side, indicating that soft tissue can be cultured adjacent to mineralized tissue (Figure 3B). A side view of the scaffold was obtained to ascertain if cells were present in the central nanofibrous region (Figure 3C). Although few cells could be seen in this barrier region, these images were hard to interpret so further analysis using fluorescent imaging was performed to confirm this.

**Figure 4:**
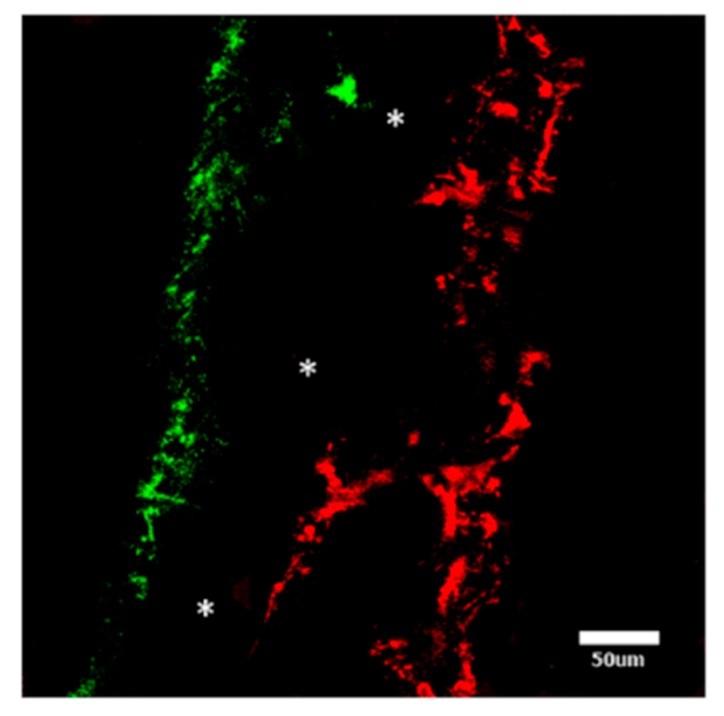
Tri-layer scaffold after 28 days culture, showing HDFs (green) and matrix mineralization by hBMSCs (red), with the nanofibre layer as an unstained region (white asterisks) in the center (scale bar = 50 µm).

Fluorescence imaging (Figure 4) revealed mineral deposition (red) on only one side of the scaffold and fibroblasts (green) only on the other, confirming the SEM images. The absence of staining in the central nanofibrous layer confirmed neither fibroblasts nor mineral depositing cells had penetrated this region.

**Figure 5:**
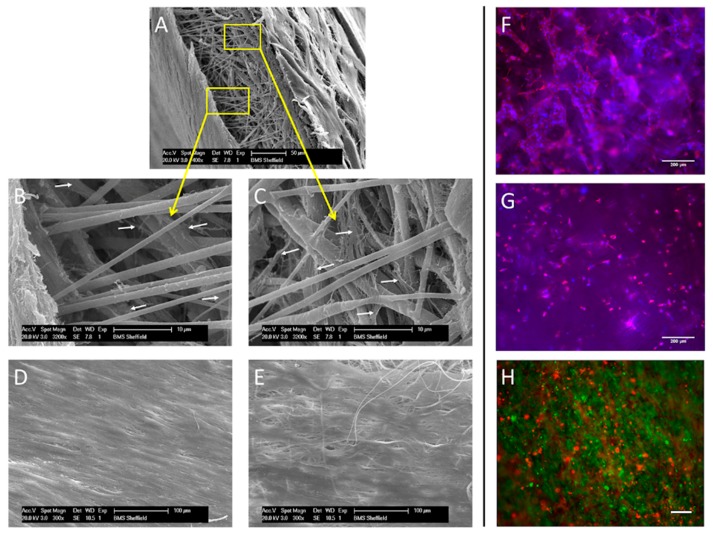
SEM side (**A**–**C**) and face views (**D**,**E**) showing the top (**B**,**D**) and bottom (**C**,**E**) of a monolayer microfiber PCL scaffold seeded on the top face only with hES-MPs after 28 days culture. Cells (white arrows) can be seen throughout the scaffold (**B**,**C**) and as sheets covering both outer surfaces (**D**,**E**), (**A**—scale bar = 50 µm; **B**,**C**—scale bar = 10 µm; **D**,**E**—scale bar = 100 µm) hES-MPs seeded on the top surface can also be detected by immunolabeling on the top (**F**) and bottom (**G**) surfaces of a microfiber PCL scaffold (scale bar = 200 µm). The images show cell nuclei (blue) and actin filaments (red) on both surfaces. Co-seeding of fibroblasts (green) and hES-MPs (red) on opposing sides of a similar scaffold results in intermingling of cells at the end of the culture period ((**H**), scale bar = 50 µm).

hES-MPs seeded on one side of a monolayer PCL microfiber scaffold, fabricated without a nanofiber barrier layer, were found to infiltrate throughout the whole scaffold after 28 days culture (Figure 5A–E). Cell infiltration throughout a microfiber PCL scaffold was also confirmed by immunolabeling of cells (Figure 5F,G). Furthermore, initial experiments with hES-MPs and fibroblasts seeded on opposing sides of a PCL microfiber scaffold demonstrated a monolayer microfiber scaffold was unable to maintain spatial segregation over the course of the culture period, resulting in the intermingling of the two cell types (Figure 5H). Previous experiments from our laboratory using PLA/PHBV [15] or PCL [16] electrospun microfiber scaffolds have also shown that fibroblasts and MSCs fail to form two distinct spatial segregated layers in the absence of this nanofiber barrier layer, with both cell types found in the same region. Furthermore the culture of MSC cells on a PCL scaffold without the barrier layer resulted in mineralization being detected throughout the scaffold [16]. Our results showed no cell infiltration or mineralization occurs within this nanofiber barrier layer in the tri-layer scaffold.

Fibroblast proliferation and MSC proliferation and mineralization were used as *in vitro* endpoints of successful soft tissue and bone regeneration. However further studies will be required to confirm that the scaffold will support full bone regeneration and soft tissue growth *in vivo* to determine the suitability of the scaffold for future clinical use.

We propose that this tri-layered scaffold could be appropriate for composite tissue engineering in a clinical procedure, particularly where bone defects are associated with defects in soft tissue. This could be achieved, e.g., with the implantation of a scaffold seeded with autologous BMSCs and buccal fibroblasts [17] on opposing faces for the treatment of cleft palate. The current practice is to repair the soft tissue defect first before addressing the bone defect. However, a scaffold that provides the opportunity to repair both defects simultaneously may have clinical benefits, including potentially reducing the time taken to repair the defects, reducing the number of operations required or improving the outcome for the patient. The successful regeneration of epithelialized soft tissues such as skin and oral mucosa on electrospun scaffolds has been demonstrated *in vitro* in our laboratory [17,18] and these techniques could be also be employed to regenerate the soft tissue on this tri-layer scaffold. In addition antibiotics could be incorporated into the electrospun scaffold [19,20] to help prevent bacterial colonization during tissue regeneration.

Although adult hBMSCs were used in this study, we expect that iliac crest MSCs derived from a child would behave in a similar manner, and may form bone more rapidly, as the osteogenic potential of hBMSCs decreases with age [21]. Other sources of osteoprogenitor cells could be employed that avoid the need for bone marrow harvest, such as adipose derived mesenchymal stem cells or periosteal cells [1,5]. We recently reported a method of electrospinning tri-layer PHBV/PLA scaffolds for the spatially segregated co-culture of keratinocytes and fibroblasts [15]. However, this scaffold is likely to be sub-optimal for clinical use with bone-forming tissue, due to the six-month degradation rate of PLA *in vivo* [14,22]. Poly-ε-caprolactone (PCL) is FDA approved and can generate biocompatible and porous electrospun scaffolds, with suitable mechanical properties to support both soft tissue and bone growth. It also has a relatively slow degradation rate compared to other commonly used biodegradable polymers [23,24]. Therefore, a PCL membrane is likely to maintain the initial fibrous structure and have sufficient mechanical strength for at least the first six months of the tissue regeneration process [2].

## 4. Conclusions

The strategy presented in this proof-of-concept study was to generate a tri-layer scaffold composed of a nanofiber barrier layer between the two microfiber layers to support both soft tissue and bone formation while maintaining spatial segregation of the two tissues. Although the results presented are preliminary, they indicate that an electrospun PCL tri-layer scaffold can be generated reproducibly that can support the fully segregated co-culture of hBMSCs cells and fibroblasts for at least 28 days and allow osteogenic differentiation with demonstrated calcium deposition. This tri-layer membrane may also be useful for other clinical situations that involve more than one type of tissue, such as guided tissue regeneration for periodontal surgical treatment, alveolar ridge augmentation for tooth implantation and surgical reconstruction following trauma or tumor removal.
